# Management of Bilateral Endogenous Methicillin-Sensitive Staphylococcus aureus (MSSA) Endophthalmitis Without Intravitreal Sampling or Injection

**DOI:** 10.7759/cureus.99807

**Published:** 2025-12-21

**Authors:** Jordan Stewart, Shoaib Hassan, Ayad Al-Bermani

**Affiliations:** 1 Department of General Surgery, University Hospital of Wales, Cardiff, GBR; 2 Department of Ophthalmology, University Hospital of Wales, Cardiff, GBR

**Keywords:** endogenous endophthalmitis, endophthalmitis, intraocular infection, intravitreal injections, methicillin-sensitive staphylococcus aureus

## Abstract

We report a 70-year-old woman who awoke with sudden, painless bilateral vision loss. Examination revealed dense vitritis, and systemic evaluation found methicillin‑sensitive *Staphylococcus aureus* septicemia secondary to lumbar facet joint septic arthritis. She received systemic intravenous flucloxacillin without intravitreal sampling or intravitreal antibiotic injection. The ocular inflammation largely resolved, and visual acuity improved to 6/6 in one eye and 6/24 in the other with systemic therapy alone. This case highlights the importance of a thorough systemic workup; blood cultures identify the causative organism in the majority of endogenous cases, whereas ocular fluid cultures yield no growth less frequently. Our experience suggests that when the systemic source of infection is identified and ocular signs are improving, a conservative approach without routine intravitreal tap and inject on presentation may be reasonable. However, treatment should be individualized, and further studies are needed to define the role of intravitreal intervention in endogenous endophthalmitis.

## Introduction

Endogenous endophthalmitis is a rare but devastating ocular infection caused by hematogenous seeding of microorganisms into the eye. In contrast to exogenous endophthalmitis, which follows trauma or surgery, endogenous cases occur when pathogens enter the ocular circulation from a distant focus of infection [1]. Because of its rarity, the condition accounts for only 2%-8% of all cases of endophthalmitis, with bilateral involvement in 15%-20% of these cases [2]. Risk factors include systemic illnesses (diabetes, malignancy, and immunosuppression), invasive medical procedures, indwelling catheters, and intravenous drug use. The microbiology varies geographically, with gram-positive bacteria such as *Staphylococcus aureus* and Streptococcus species being common causes in the Western world.

Diagnosis relies on ocular findings, absence of ocular trauma/surgery, and identification of a systemic focus of infection. In approximately 90% cases, the causative organism is identified through blood, urine, or cerebrospinal fluid (CSF) cultures. Many guidelines recommend a "tap and inject" strategy for endophthalmitis, which entails vitreous biopsy and intravitreal antibiotics upon diagnosis. However, given the high yield of systemic cultures and the potential risks of intraocular procedures, some authors advocate a more selective approach in endogenous cases, reserving intravitreal injections for cases with persistent infection, lack of systemic organism identification, or rapid ocular deterioration. Multiple guidelines also suggest a six-week course of antibiotics, which may be appropriate alone in some cases. This particular case illustrates this dilemma [3,4].

## Case presentation

Patient and presentation

We report a 70-year-old woman with Parkinson’s disease, living independently with no prior ocular pathology. She awoke with sudden, painless bilateral visual loss; the day before, she had normal vision. Four days earlier, she had experienced an unwitnessed fall associated with approximately seven hours of unconsciousness, but did not seek medical evaluation after contacting the emergency telephone service.

Initial assessment

**Figure 1:**
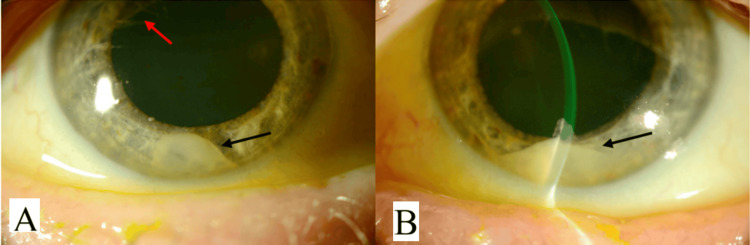
Views of the anterior chamber of both eyes upon slit lamp examination. (A) Right eye. (B) Left eye Black arrows show hypopyon in the right and left eyes. Posterior synechiae (red arrow) are visible in the right eye

**Figure 2:**
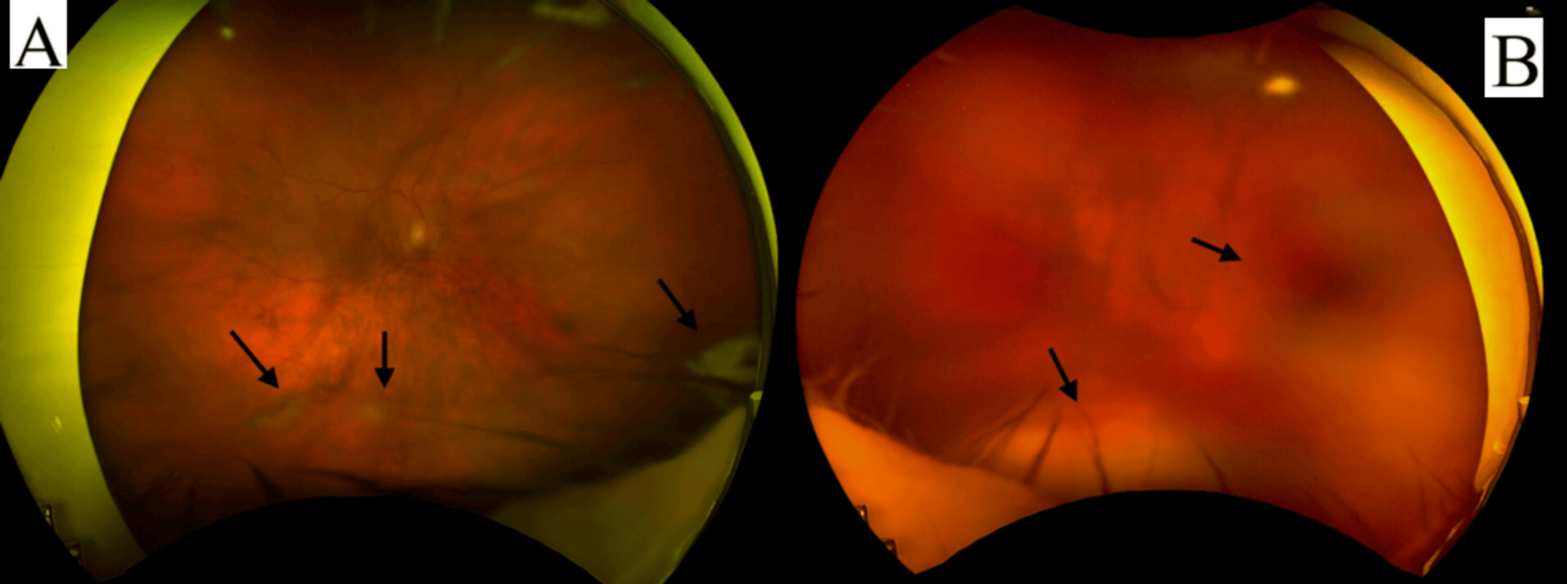
Vitreous veiling and areas of active inflammation (black arrows). (A) Right eye. (B) Left eye

On initial examination, her visual acuity was limited to counting fingers without improvement on pinhole, having previously been 6/6 in both eyes. Slit lamp examination showed dense anterior chamber inflammation with hypopyon, flare, and posterior synechiae bilaterally (Figure 1). The fundus view was obscured by dense vitreous opacities (Figure 2), with B-scan ultrasonography further demonstrating dense vitritis, more prominent on the left. In the absence of ocular surgery and given the rapid progression, endogenous endophthalmitis was suspected. Systemic evaluation revealed markedly elevated inflammatory markers (C‑reactive protein 375 mg/L, white cell count 34.3 × 10⁹ cells/L). Blood cultures became positive within 10 hours, yielding gram‑positive cocci, subsequently identified as methicillin-sensitive *Staphylococcus aureus*.

Imaging

**Figure 3:**
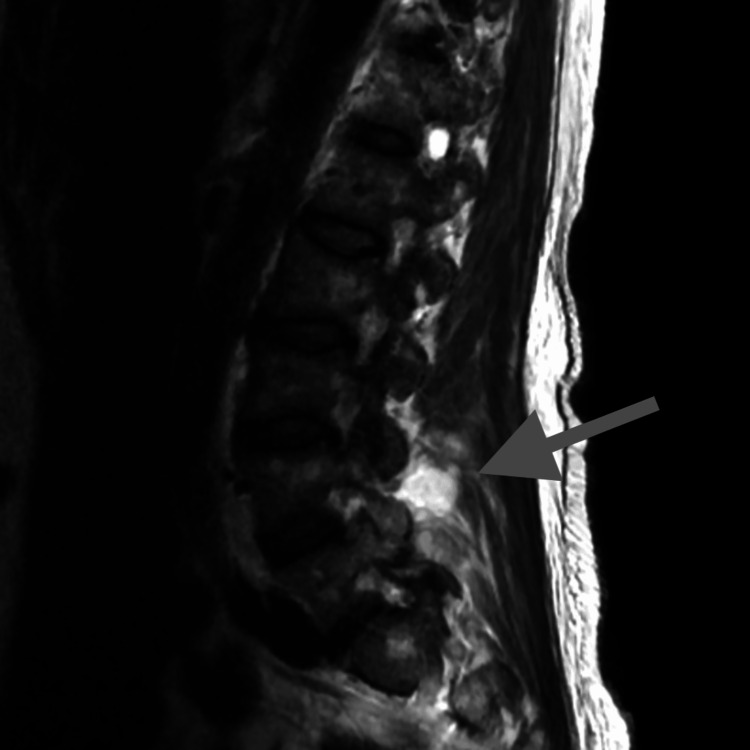
Area of increased signal intensity near L4/5 facet joint (see the gray arrow) due to local inflammation

CT head on admission was unremarkable. After the causative organism was identified, further examination of the patient's lumbar spine revealed tenderness at the L4 level. Subsequent MRI of the lumbar spine (Figure 3) showed septic arthritis of the L4/5 facet joints with extension into the epidural space, indicating the likely source of infection.

Management and outcome

The patient was admitted to the medical team for systemic management and began empirical intravenous flucloxacillin. Topical prednisolone acetate 1% and cyclopentolate 1% drops were started three times daily in both eyes. Because the causative organism was identified early from blood cultures, systemic indices were improving, and there was no ocular deterioration, the team elected not to perform vitreous or aqueous sampling, and no intravitreal antibiotics were given. The L4/5 facet joint was drained under ultrasound guidance, and infection markers continued to improve until discharge.

Visual acuity improved to 6/24 in the right eye and counting fingers in the left by the second week. After three weeks, she was discharged on a six-week course of oral moxifloxacin and adjunctive rifampicin, indicated by culture sensitivities. Moxifloxacin was selected due to its superior penetration into the posterior chamber compared to other fluoroquinolones. Rifampicin was selected as an adjunct for broader coverage following facet joint aspiration. At follow-up one week after discharge, her best corrected visual acuity was 6/6 in the right eye and 6/24 in the left eye. On examination, there was significant clearing of vitreous debris, resolving hypopyon and posterior synechiae, with complete resolution of her systemic symptoms. She continued on another six-week course of antibiotics, and at 12 weeks, visual acuity had been restored to baseline in both eyes. The patient was able to continue with her daily activities and drive without any issues.

## Discussion

Bilateral endogenous endophthalmitis (EE) is an exceptionally rare entity associated with substantial long-term morbidity, including profound vision loss and even mortality [2,5]. EE poses a diagnostic and therapeutic challenge because it arises from systemic infection and often presents without obvious ocular trauma or surgery. In this patient, the source was identified promptly, which undoubtedly contributed to the positive outcome. It is important to note that, as with the majority of EE cases, the source was identified through means other than intravitreal sampling. Evidence indicates that ocular fluid cultures have variable and often low yields. In a two-decade retrospective analysis, infection positivity rates were 70% for blood cultures alone compared with 54% for vitreous samples of the same cohort [5-7]. Vitrectomy samples yield positive cultures in 44%-90% of cases [8]. In contrast, systemic cultures (blood, urine, CSF) identify the pathogen in a majority of endogenous cases [5,6]. These findings support a strategy of reserving intraocular sampling for situations where systemic cultures are negative, where there is no clinical improvement or when ocular involvement rapidly progresses.

Many centers worldwide recommend prompt vitreous sampling and intravitreal administration of broad-spectrum antibiotics, along with systemic therapy for EE [5,9]. The rationale is to achieve high intraocular antimicrobial concentrations and to obtain specimens to guide treatment. However, intravitreal procedures carry risks, including retinal detachment, hemorrhage, and acceleration of inflammation [9]. Our patient demonstrated significant ocular and systemic improvement with systemic antibiotics alone. This aligns with emerging literature suggesting that selected patients with an identifiable systemic source of infection and improving ocular signs may be managed without intravitreal tap and inject [9]. Furthermore, the use of newer fluoroquinolone antibiotics with better ocular penetrance, such as moxifloxacin (used in this case), may further diminish the need for "tap and inject" strategies for EE [9]. Nevertheless, caution is warranted: systemic antibiotics alone may be insufficient in severe or rapidly progressive cases [5,9], particularly when the causative organism is unknown or when fungal infection is suspected. The variability in reported culture yields and outcomes has led to heterogeneity in clinical practice. In many centers, intravitreal sampling remains routine for endogenous endophthalmitis, yet evidence supporting a universal “tap and inject” strategy is limited [9]. Some retrospective series report vision-preserving outcomes with systemic therapy alone, while others highlight the benefits of early vitrectomy. The case presented here demonstrates that individualized management, guided by systemic culture results, ocular findings, and response to therapy, can lead to favorable outcomes without intraocular intervention. Prospective studies are needed to develop evidence-based algorithms to determine the threshold for intravitreal sampling and antibiotics.

## Conclusions

Endogenous endophthalmitis is rare, yet it is potentially sight-threatening. The majority of cases have positive systemic cultures, and blood cultures also identify the pathogen in the majority of patients. Negative culture rates are high for aqueous and vitreous taps; therefore, intravitreal sampling at presentation may be unnecessary in selected patients when the systemic source is identified and ocular inflammation is improving. Consensus based on prospective data is needed to demonstrate this further; however, care should always be guided by patient factors and specialist opinion.
